# Pretreatment with a dual antiplatelet and anticoagulant (APAC) reduces ischemia–reperfusion injury in a mouse model of temporary middle cerebral artery occlusion—implications for neurovascular procedures

**DOI:** 10.1007/s00701-024-06017-x

**Published:** 2024-03-15

**Authors:** Frederik Denorme, Juhana Frösen, Annukka Jouppila, Antti Lindgren, Julio C. Resendiz-Nieves, Hannu Manninen, Simon F. De Meyer, Riitta Lassila

**Affiliations:** 1 Laboratory for Thrombosis Research, KU Leuven Campus Kulak Kortrijk, Kortrijk, Belgium; 2 Hemorrhagic Brain Pathology Research Group, Dept. of Neurosurgery, Kuopio University Hospital, Kuopio, Finland; 3 Department of Neurosurgery, Tampere University Hospital, Tampere, Finland; 4 Helsinki University Central Hospital Clinical Research Institute, Helsinki, Finland; 5 Department of Clinical Radiology, Kuopio University Hospital, Kuopio, Finland; 6 Institute of Clinical Medicine, School of Medicine, Faculty of Health Sciences, University of Eastern Finland, Kuopio, Finland; 7 Department of Neurosurgery, University of Helsinki and Helsinki University Hospital, Helsinki, Finland; 8 Department of Radiology, Kuopio University Hospital, Kuopio, Finland; 9 Coagulation Disorders Unit, Departments of Hematology and Cancer Center, Helsinki University Hospital, Helsinki, Finland; 10 Faculty of Medicine, Research Program in Systems Oncology, University of Helsinki, Helsinki, Finland; 11 Aplagon Oy, Helsinki, Finland

**Keywords:** Antiplatelet, Anticoagulant, APAC, Infarct, Ischemia–reperfusion injury, Transient, Cerebral ischemia

## Abstract

**Background:**

Several neurovascular procedures require temporary occlusion of cerebral arteries, leading to ischemia of unpredictable length, occasionally causing brain infarction. Experimental models of cerebral ischemia–reperfusion injury have established that platelet adhesion and coagulation play detrimental roles in reperfusion injury following transient cerebral ischemia. Therefore, in a model of cerebral ischemia–reperfusion injury (IRI), we investigated the therapeutic potential of a dual antiplatelet and anticoagulant (APAC) heparin proteoglycan mimetic which is able to bind to vascular injury sites.

**Methods:**

Brain ischemia was induced in mice by transient occlusion of the right middle cerebral artery for 60 min. APAC, unfractionated heparin (UFH) (both at heparin equivalent doses of 0.5 mg/kg), or vehicle was intravenously administered 10 min before or 60 min after the start of ischemia. At 24 h later, mice were scored for their neurological and motor behavior, and brain damage was quantified.

**Results:**

Both APAC and UFH administered before the onset of ischemia reduced brain injury. APAC and UFH pretreated mice had better neurological and motor functions (*p* < 0.05 and *p* < 0.01, respectively) and had significantly reduced cerebral infarct sizes (*p* < 0.01 and *p* < 0.001, respectively) at 24 h after transient occlusion compared with vehicle-treated mice. Importantly, no macroscopic bleeding complications were observed in either APAC- or UFH-treated animals. However, when APAC or UFH was administered 60 min after the start of ischemia, the therapeutic effect was lost, but without hemorrhaging either.

**Conclusions:**

Pretreatment with APAC or UFH was safe and effective in reducing brain injury in a model of cerebral ischemia induced by transient middle cerebral artery occlusion. Further studies on the use of APAC to limit ischemic injury during temporary occlusion in neurovascular procedures are indicated.

**Supplementary Information:**

The online version contains supplementary material available at 10.1007/s00701-024-06017-x.

## Introduction

Neurovascular interventions, whether endovascular or microsurgical, carry a risk of vessel occlusion and may lead to ischemia, and at worst, to an established infarction [11, 17]. While in many instances the vessel occlusion can be reopened, by medical or mechanical means, ischemic injury may still ensue [11, 17]. Ischemic brain injury may also occur due to temporary cerebral artery occlusion, frequently used in endovascular procedures to treat aneurysms, for example, an intra-arterial balloon to properly position coils inside the aneurysm sac, as well as in cerebrovascular microsurgery, for example, to decompress an intracranial aneurysm before microsurgical ligation [20, 24], or to limit hemorrhage in the event of an intra-operative rupture (Supplemental Fig. 1) [14]. Although the aim is to minimize the duration of such a temporary occlusion, it is difficult to predict the duration of this kind of intervention in advance. Sometimes completing the procedure successfully requires prolonged temporary occlusion longer than what is generally considered safe. Any measure that can potentially promote the survival of brain tissue under transient ischemia and limit the extent of subsequent ischemia–reperfusion injuries would likely improve patient outcomes in neurovascular interventions and also increase their safety.

The phenomenon of cerebral ischemia–reperfusion injury has extensively been studied in murine stroke models [5]. From these studies, it has become clear that once reperfusion of the ischemic brain is initiated, a detrimental cascade of coagulation, platelet adhesion, and inflammation converge causing thrombo-inflammatory brain injury. This phenomenon plays an important role in the exacerbation of ischemic brain injury despite the restoration of blood flow. Inhibition of coagulation or platelet adhesion has been shown to limit the extent of brain damage due to transient ischemia [5].

Heparin is an antithrombotic that carries anticoagulant behavior through its binding and activation of antithrombin. Heparin is derived from mast cells, which line the vascular wall. Upon tissue injury, mast cells are activated, and release heparin proteoglycans (HEP-PGs), which are much higher in molecular weight than the therapeutically used unfractionated heparin (UFH). Interestingly, HEP-PGs have been shown to exhibit both anticoagulant features, as does heparin typically, as well as specific antiplatelet properties, most notably involving platelet-collagen interactions [3, 13]. Because of these interesting properties, semi-synthetic antiplatelet and anticoagulant (APAC) heparin proteoglycan mimics have gained interest as therapeutics for thrombotic diseases [3, 13]. In vitro, APAC concentration-dependently inhibits collagen- and thrombin-induced platelet aggregation both in human and murine platelet-rich plasma (PRP) and reduces both platelet deposition and fibrin formation on thrombogenic surfaces under arterial shear rate conditions in vitro and in vivo models [2, 3, 13, 22, 23]. In vivo, it was shown that locally administered APAC binds to the vascular injury site and resides there to exert its antiplatelet and anticoagulation properties [1, 2, 13]. Inhibition of thrombus growth using APAC under these conditions has already been demonstrated in several animal models, including baboons [2, 13]. Specifically, two distinct mouse models showed the targeting ability of APAC (IV, 0.5 mg/kg) with reduced platelet deposition at the collagen-rich arterial injury sites and delayed arterial occlusion at photochemically-induced carotid artery thrombosis (IV, 0.3 mg/kg) [2]. Also, a smaller prophylactic dose of APAC (IV, 0.13 mg/kg) protected from ischemic reperfusion injury (IRI) in a rat model of acute kidney injury [22]. Importantly, while APAC was more potent in preventing pathological thrombus formation, bleeding times were shorter with APAC, compared with UFH [13]. This advantage of APAC over UFH could increase safety in endovascular procedures that require heparinization. Moreover, the dual antithrombotic property of APAC is likely more beneficial than UFH under situations when focal platelet inhibition and anticoagulation are needed during open surgery.

In this study, we assessed the therapeutic potential of APAC and UFH (0.5 mg/kg, UFH at 90 IU/kg) in a well-established mouse model of transient cerebral ischemia/reperfusion injury. APAC dose was selected based on the previous in vivo potency in several animal models, including the safety toxicology [2, 4, 13, 22]. We found that APAC was protective when administered before cerebral ischemia but lost its protective effect when treatment was delayed until the reperfusion phase. Similar protective data by APAC but not by UFH have been reported in acute ischemic kidney injury [22]. Our results suggest that APAC may be useful to limit the extent of IRI under situations with the known onset of transient ischemia, for instance in temporary vessel occlusion during endovascular or microsurgical procedures (Supplemental Figs. 1, 2 and 3).

## Materials and methods

### Mice and treatment

All animal studies were performed in accordance with the guidelines of the local ethical committees (KU Leuven, Leuven, Belgium; act no. 87–848) and following the ARRIVE guidelines (www.nc3rs.org.uk), including randomization of treatment as well as surgery and analysis blinded to the treatment. Mice (*n* = 80) used in these experiments were 8- to 10-week-old male and female C57BL/6 mice (The Jackson Laboratory, Bar Harbor, ME, USA). Treatment was administered IV by injection into the retro-orbital venous sinus (retro-orbital injection) 10 min before or 60 min after ischemic stroke onset. Treatment consisted of either 0.5 mg/kg APAC (stock solution 7.15 mg/ml, 10 mM Na_2_HPO_4_, 137 mM NaCl, pH 7.5, Aplagon Ltd., Helsinki, Finland) or 0.5 mg/kg (~ 90 IU/kg) heparin (UFH, Leo Pharma, Ballerup, Denmark) or control (0.9% NaCl).

### Cerebral ischemia and reperfusion injury model

The middle cerebral artery (MCA) was transiently occluded (tMCAO), as described previously [6]. Anesthesia was induced by inhalation of 5% isoflurane and maintained by inhalation of 2% isoflurane. After a midline incision in the neck, the proximal common carotid artery and the external carotid artery were ligated, and standardized silicon rubber-coated 6–0 nylon monofilament (6021; Doccol Corp, Redlands, CA) was inserted and advanced via the right internal carotid artery to occlude the origin of the right MCA. The intraluminal suture remained in place for 60 min, during which time the mice were permitted to regain consciousness. Successful stroke induction was confirmed by neurological scoring, as described below. Subsequently, the animals were re-anesthetized, and the occlusive monofilament was removed to enable reperfusion. The duration of each surgical procedure for each animal did not surpass 10 min. The following criteria excluded mice from the endpoint analyses: death within 24 h after tMCAO or the occurrence of subarachnoid hemorrhaging due to surgical complications. Of the 80 mice subjected to tMCAO, four were excluded because they died during the time of the experiment, and seven mice were excluded due to subarachnoid hemorrhaging. No difference in mortality rate or subarachnoid hemorrhaging was observed between the different experimental groups. Researchers and operators were blinded to treatment and analysis for all readout parameters, with unblinding occurring only after all analyses were complete.

### Neurological tests

Twenty-four hours after induction of tMCAO, the mice were subjected to the modified Bederson test and the grip test to assess global neurological and motor function, respectively [22]. The modified Bederson test uses the following scoring system: 0 = no deficit; 1 = forelimb flexion; 2 = decreased resistance to lateral push; 3 = unidirectional circling; 4 = longitudinal spinning; and 5 = no movement. During the grip test, a mouse is placed on a wooden bar attached to 2 vertical supports 40 cm above a flat surface. When placing the mouse on the bar midway between the supports, the experiment was rated according to the following system: 0 = the mouse falls off; 1 = hangs onto bar by 2 forepaws; 2 = same as for 1, but attempts to climb onto bar; 3 = hangs onto bar by 2 forepaws plus 1 or both hind paws; 4 = hangs onto bar by all 4 paws plus tail wrapped around bar; and 5 = escape (mouse is able to reach one of the supports).

### Measurement of infarct volume

Mice were euthanized at 24 h after occlusion of the MCA. Brains were removed and cut into 2-mm-thick coronal sections. Brain sections were stained with 2% 2,3,5-triphenyl-tetrazolium chloride (TTC, T8877, Sigma-Aldrich, Saint-Louis, MO, USA) in phosphate buffered saline (PBS) to visualize healthy tissue (pink) and unstained infarctions (white). Sections were photographed, and infarct areas (*V*_uncorrected_) were analyzed via planimetry using ImageJ software (National Institutes of Health, Bethesda, MD; http://imagej.nih.gov/ij/) by an experimenter, who was blinded for the treatment conditions. Edema-corrected infarct sizes (*V*_corrected_) were calculated using the following equation: *V*_corrected_ = *V*_uncorrected_ × (1 − (*V*_*i*_ − *V*_*c*_)/*V*_*c*_) where *V*_*i*_ is the volume of the ipsilateral hemisphere and *V*_*c*_ is the volume of the contralateral hemisphere. The presence of cerebral hemorrhages was macroscopically assessed upon brain isolation, as well as via careful visual analysis of the coronal brain sections after cutting.

### Assessment of the anticoagulant activity in mouse plasma in vitro

The anticoagulant activity of spiked (0.25–1 µg/ml) APAC and UFH was confirmed in (citrated, 3.8%) mouse plasma (C57BL/6, Innovative Research, Novi, MI, USA) with calibrated automated thrombogram (CAT) analysis, using 5 pM of tissue factor to trigger the thrombin generation (TG) according to manufacturer’s instructions (CAT System, Diagnostica Stago, Asnieres sur Seine Cedex, France). Thrombin time (TT) (STA-Thrombin 2, Diagnostica Stago) and activated partial thromboplastin time (APTT) (SynthAFax, HemosIL, Instrumentation Laboratory, Bedford, MA, USA) were assessed using STart Max analyzer (Diagnostica Stago) at a concentration range of 0.25–2 µg/ml of APAC or UFH [2]. The maximal detection time for TT and APTT was 120 s.

### Statistical analysis

All data are presented as mean and standard deviation (SD), except for the Bederson and the grip test scores, which are depicted as scatter plots, including the median with the interquartile range (IQR). Statistical analysis was performed with GraphPad Prism Version 6.0e. The number of experimental animals in each group was based on power calculations with infarct volume as the primary parameter and with mean differences and standard deviations obtained from available data from the same stroke models performed earlier (power of 80% and *α* of 0.05). Prior to statistical analysis, a D’Agostino and Pearson test were applied to check data distribution. An ordinary one-way ANOVA corrected for multiple comparisons using a Dunnett test, or a Mann–Whitney test with post hoc Dunn correction was used for statistical comparison of lesion volumes where appropriate. In the case of non-parametric data (Bederson and grip test scores), a Kruskal–Wallis test with post hoc Dunn correction was performed.

## Results

### Pretreatment with APAC or UFH reduces cerebral ischemia/reperfusion injury without introducing cerebral hemorrhaging

**Fig. 1:**
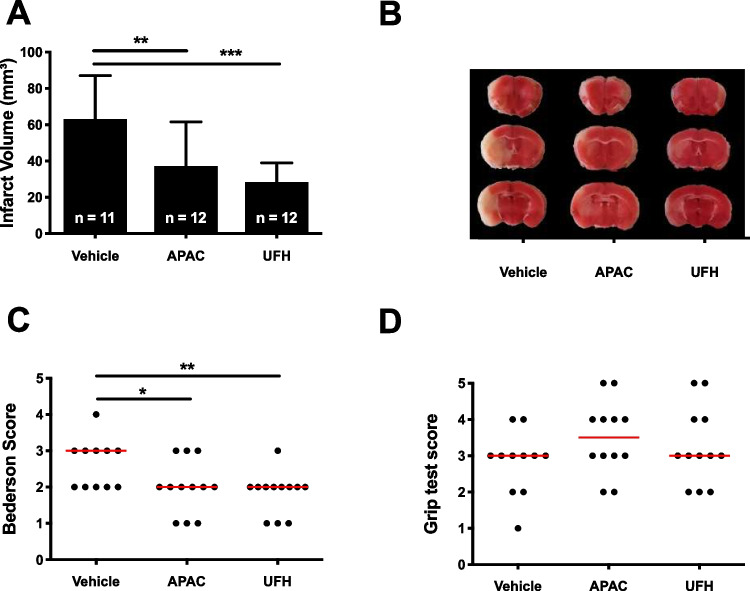
Effect of APAC and UFH pretreatment on cerebral ischemia–reperfusion damage in mice. **A** Edema-corrected brain infarct volumes were quantified by planimetric analysis. A one-way ANOVA corrected for multiple comparisons using a Dunnett test was used to compare experimental mice with saline-treated mice. **B** Representative 2,3,5-triphenyl tetrazolium chloride (TTC)-stained brain sections. Healthy tissue stains red, while the absence of staining (white) indicates the infarcted area. **C** Neurological outcome was assessed using the Bederson test. **D** Motor function was examined using the grip test. For these functional tests, a Kruskal–Wallis test with post hoc Dunn correction was used to compare the experimental mice with control-treated mice

Using a stroke model of tMCAO, mice were treated 10 min before the start of ischemia with APAC, UFH, or vehicle. At 24 h later, brains were isolated to determine infarct volumes (mean ± SD; Fig. 1A, B). Vehicle-treated mice developed infarct sizes of 63 ± 24 mm^3^. In mice treated with APAC or UFH, the brain injury was significantly smaller. APAC-treated mice had infarcts that were 40% smaller compared to vehicles (37 ± 24 mm^3^; *p* < 0.01). Similarly, UFH treatment led to a 50% reduction in infarct volumes (28 ± 10 mm^3^; *p* < 0.001). APAC or UFH treatment had a similar effect (*p* > 0.05). Importantly, visible bleeding complications were not observed in vehicle-treated mice, neither in APAC- nor in UFH-treated mice.

In agreement with the smaller brain lesions, both APAC and UFH treatment led to better neurological outcomes (Fig. 1C). Neurological outcome, as assessed by the Bederson score, was significantly improved for both APAC- (2 (1.25, 2.75) as median (25th and 75th percentiles); *p* < 0.05) and UFH- (2 (1.25, 2); *p* < 0.01) treated mice compared to vehicle-treated mice (3 (2, 3)). The motor outcome was assessed by the grip test, without significant improvement in the treatment groups (Fig. 1D), as mice receiving APAC (3.5 (3, 4)), UFH (3 (2.25, 4)), or vehicle (3 (2, 3)) treatment all scored similarly.

### Protective effect of APAC or UFH is lost when the treatment is delayed

**Fig. 2:**
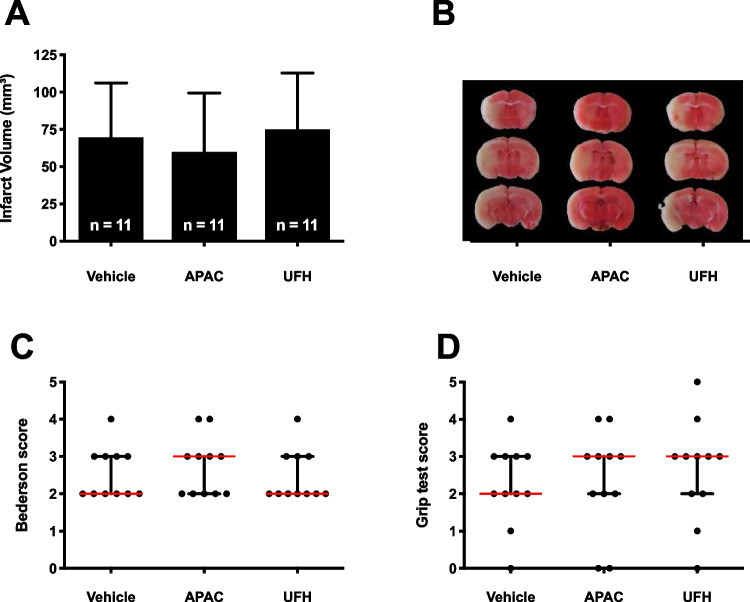
Effect of delayed APAC and UFH treatment on cerebral ischemia–reperfusion damage in mice. **A** Edema-corrected brain infarct volumes were quantified by planimetric analysis. A Mann–Whitney test with post hoc Dunn correction was used to compare experimental mice with saline-treated mice. **B** Representative 2,3,5-phenyl TTC-stained brain sections. Healthy tissue stains red, while the absence of staining (white) indicates the infarcted area. **C** Neurological outcome was assessed using the Bederson test. **D** Motor function was examined using the grip test. For these functional tests, a Kruskal–Wallis test with post hoc Dunn correction was used to compare experimental mice with control-treated mice

In the second set of experiments, treatment was given after 1 h of tMCAO, thus at the start of initiating reperfusion. At 24 h after stroke onset, brains were isolated to determine infarct volumes (mean ± SD; Fig. 2A, B). In this setting, infarct sizes were similar between APAC- (60 ± 40 mm^3^), UFH- (75 ± 43 mm^3^), and vehicle- (70 ± 37 mm^3^) treated mice. Correspondingly, Bederson ((3 (2, 3)); (2 (2, 3)); (2 (2, 3)); *p* > 0.05; Fig. 2C) and grip test scores ((3 (2, 3)); (3 (2, 3)); (2 (2, 3)); *p* > 0.05; Fig. 2D) were equal for APAC, UFH, and vehicle treatment groups, respectively. No bleeding complications were observed in any of the mice.

### Anticoagulant activity of APAC and UFH in mouse plasma in vitro

**Fig. 3:**
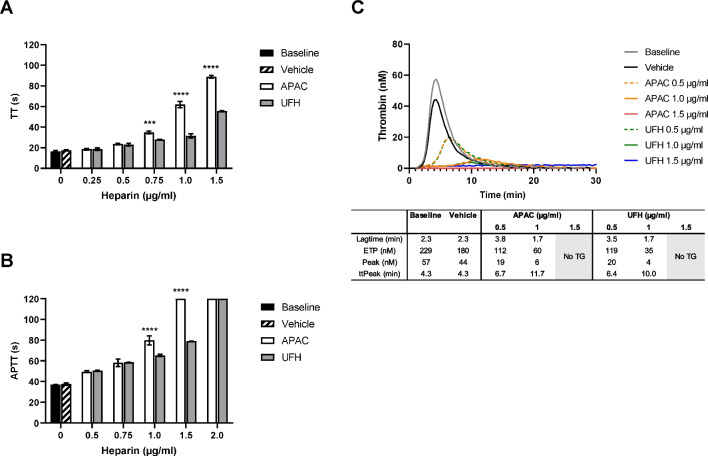
Anticoagulant activity of APAC and UFH in mouse plasma in vitro. APAC and UFH concentration-dependently prolonged **A** TT and **B** APTT and **C** reduced and delayed TG by CAT in APAC- or UFH-spiked 3.8%-citrated mouse plasma. Coagulation times: ***p < 0.001, ****p < 0.0001, *n* = 3–4, and mean ± SD. The maximal detection of coagulation time was 120 s. CAT was analyzed once. Lag time, time to initiation of TG; ETP, endogenous thrombin potential; peak, maximum TG; ttpeak, time to peak

APAC and UFH prolonged coagulation times (TT and APTT) in mouse plasma concentration-dependently at above 0.5 µg/ml (Fig. 3A, B). At 0.75–1.5 µg/ml, APAC prolonged TT 20 to 50% more than UFH (*n* = 3, *p* < 0.0001; Fig. 3A). Also, at 1.0 µg/ml, APAC was 20% more potent anticoagulant in APTT than UFH, and at 1.5 µg/ml, APAC prolonged APTT up to the maximal detection time (*n* = 3–4, *p* < 0.0001; Fig. 3B). In TG analysis, both APAC and UFH inhibited the exogenous TF-triggered thrombin potential and maximal TG and prolonged the lag time and time to peak values, in comparison to control (Fig. 3C).

## Discussion

Our study demonstrates that APAC or UFH when administered before the onset of transient occlusion of an intracranial artery efficiently reduces the development of IRI and subsequent brain infarction. Moreover, in our model, APAC and UFH were safe and did not promote intracerebral hemorrhage, even when administered after irreversible ischemic injury had developed.

In line with our current results, we have previously observed a similar protective effect of APAC in the setting of renal ischemia/reperfusion injury, although in that setting, UFH was ineffective [22]. The mechanism through which APAC limits ischemia/reperfusion injury is likely attributed to the prevention of platelet and fibrin deposition in the ischemic tissue, as previously suggested [16]. These antiplatelet and anticoagulant properties likely prevent both macro- and microvascular stasis in the brain and result in improved cerebral blood flow during reperfusion. This hypothesis is supported by a recent study by Desilles and colleagues who visualized platelet deposition and fibrin formation in the brain using a similar ischemic stroke model [7]. An important observation from their study was the occurrence of platelet and fibrin deposition already during ischemia. This could possibly explain why APAC was no longer effective when treatment was delayed until 1 h after the onset of cerebral ischemia. APAC, when circulating at the time of vascular injury, is able to bind to the injury site to prevent platelet adhesion/activation through its antiplatelet effect [9, 13] and to inhibit thrombin/fibrin generation through its anticoagulant effect [4, 13], thus decreasing platelet and fibrin deposition at the site of ischemic insult. Also, APAC induces downstream inhibitory signalling through a platelet receptor G6b-B, an import regulator of platelet activation and production [23]. Importantly, our results suggest that APAC administered before transient occlusion could be used to limit brain injury in the context of endovascular or microvascular neurosurgical procedures (Supplemental Figs. 1, 2 and 3).

The low blood volume of mice excluded the assessment of the antithrombotic activity under the in vivo studies. We have previously shown in rats that APAC (IV 0.5–20 mg/kg) dose-dependently prolongs plasma APTT [4]. Specifically, 0.5 mg/kg of APAC prolongs APTT 2.5–fourfold of the baseline (15 min after administration), while after 90 min, the anticoagulant activity will return to baseline, the time of euthanasia in the current mouse model. We confirmed the concentration-dependent anticoagulant activity of APAC and UFH with in vitro spiking in mouse plasma and analysis of thrombin generation and coagulation times (Fig. 3).

### APAC to prevent ischemic injury caused by temporary vessel occlusion in endovascular or microsurgical procedures

Temporary occlusion of a cerebral artery to reduce pressure in an aneurysm or to control potential intra-operative bleeding of an aneurysm is an essential part of modern microsurgical operative techniques [10]. Similarly, the use of an occlusive intra-arterial balloon to properly position coils inside the aneurysm sac during embolization (Supplemental Fig. 1) and to decompress a large aneurysm during microsurgical clipping or to control for bleeding in the event of an intra-procedural rupture are all part of standard endovascular techniques [15]. Although the aim is to keep the duration of the temporary occlusion to a minimum, timely reopening of the temporary occlusion is not always possible. Extrapolating from our experimental results, administration of APAC prior to the temporary occlusion could potentially increase the tolerance of the brain to transient ischemia, and as such broaden the safety margin of cerebrovascular procedures. While perioperative use of APAC in open microsurgery could have limitations under hemorrhaging conditions, APAC treatment did not increase the risk of intracranial hemorrhaging, and we have shown that normal hemostasis is better maintained in APAC treatment compared with UFH. Having only a focal anticoagulant/antithrombotic effect [1], combined with a short duration of the systemic anticoagulant effect, could further reduce the bleeding risk. Therefore, intra-operative use of APAC in endovascular neuro-interventions seems a promising strategy. Despite these promising results, it is important to acknowledge that we did not perform a detailed histological screening of post-stroke hemorrhaging in the current study. Therefore, although we did not detect macroscopic bleeding in the APAC or heparin treatment groups, this does not exclude the possibility of microscopic bleeding. Future studies will need to evaluate the safety of APAC administration associated with the current practice.

### APAC as an alternative to UFH heparinization in neuroendovascular procedures

The main complication of neuro-endovascular procedures is ischemic events, with some studies reporting an incidence of more than 40% of ischemic lesions when evaluated by magnetic resonance imaging (MRI). Most of these events go clinically unnoticed by standard neurological exams, but that does not mean they are insignificant [8]. Furthermore, occlusion of a large cerebral artery due to perioperative thrombosis as for example within stents, or occlusion of a smaller artery due to emboli arising from the thrombosing aneurysm or damaged endothelium by the intraluminal catheters, can cause devastating brain infarctions [12, 18, 19]. To prevent thromboembolic events, UFH is administered before catheter insertion as a standard of care; however, the rate of thromboembolic complications suggests that UFH is insufficient [14, 18]. The tendency of highly negatively charged APAC to bind to exposed subendothelial surfaces, as in the case of endothelial damage caused by the intraluminal catheters, raises the possibility that APAC could prevent platelet adhesion/deposition and thrombin formation at these sites. UFH, in contrast, does not delay platelet recruitment at sites of endothelial damage [2, 3].

Additionally, under situations where APAC can be administered directly to the site prone to develop thrombosis, such as the thrombosing aneurysm neck (Supplemental Fig. 2), APAC applied locally could prevent platelet-thrombus growth and thrombin generation, limiting the in situ thrombosis and subsequent thromboembolism [2]. In our previous experimental studies, APAC interacts with the von Willebrand factor [1, 3], a key player in capturing and recruiting platelets from circulation to the injury site under arterial shear rate conditions. APAC reduces excessive platelet deposition and fibrin formation in particular under high shear stress situations of small arteries, such as brain arteries, when exposed to vascular collagen and tissue factor [3, 13]. Importantly, APAC allows better hemostasis when compared with the same dose of UFH [3, 13]. Overall, APAC could be an attractive option when treating ruptured intracranial aneurysms in the acute setting with stents and flow diversion devices which usually require the initiation of double antithrombotic medication to prevent stent thrombosis and its devastating clinical consequences [19, 21]. The local antiplatelet properties of APAC could prove useful under these situations, probably avoiding the necessity of systemic double antithrombotic therapy during the acute phase. Therein hemostasis is paramount not only to prevent aneurysm rebleeding but also to allow the performance of other rather common procedures in these patients, like ventriculostomies and spinal taps. The development of stents and flow diverters covered with APAC could serve an antithrombotic role at these sites.

## Conclusions

We demonstrate in a mouse model of transient cerebral ischemia induced by temporary occlusion of the middle cerebral artery, how pre-ischemia administered APAC appears as safe and as efficient as UFH to reduce brain infarction. This study together with our earlier observations and clinical examples implies that APAC could be useful in neuro-interventional procedures, which expose the patient to a risk of ischemic complications. Clinical safety and efficacy studies will be needed to evaluate the suitability of APAC, in these vulnerable neurointerventional procedures.

## Supplementary Information

Below is the link to the electronic supplementary material.

Supplementary file1 (PDF 467 KB)

Supplementary file2 (PDF 535 KB)

Supplementary file3 (PDF 508 KB)

## Data Availability

Original data are available upon request.

## Abbreviations

APAC: Antiplatelet and anticoagulant
APTT: Activated partial thromboplastin time
CAT: Calibrated automated thrombogram
CT: Computed tomography
HEP-PG: Heparin proteoglycan
IQR: Interquartile range
IRI: Ischemia-reperfusion injury
MCA: Middle cerebral artery
MRI: Magnetic resonance imaging
PBS: Phosphate buffered saline
Pcom: Posterior communicating artery
PRP: Platelet-rich plasma
TG: Thrombin generation
tMCAO: Transient middle cerebral artery occlusion
TT: Thrombin time
TTC: 2,3,5-Triphenyl-tetrazolium chloride
UFH: Unfractionated heparin
