# Supplementary material for: Pretreatment with a dual antiplatelet and anticoagulant (APAC) reduces ischemia–reperfusion injury in a mouse model of temporary middle cerebral artery occlusion—implications for neurovascular procedures
Source: Acta Neurochir (Wien). 2024 Mar 15;166(1):137. doi: 10.1007/s00701-024-06017-x (PMC10940479; doi:10.1007/s00701-024-06017-x)
Supplement: Supplementary file 1 — Supplementary file1 (PDF 467 KB) [file 701_2024_6017_MOESM1_ESM.pdf]

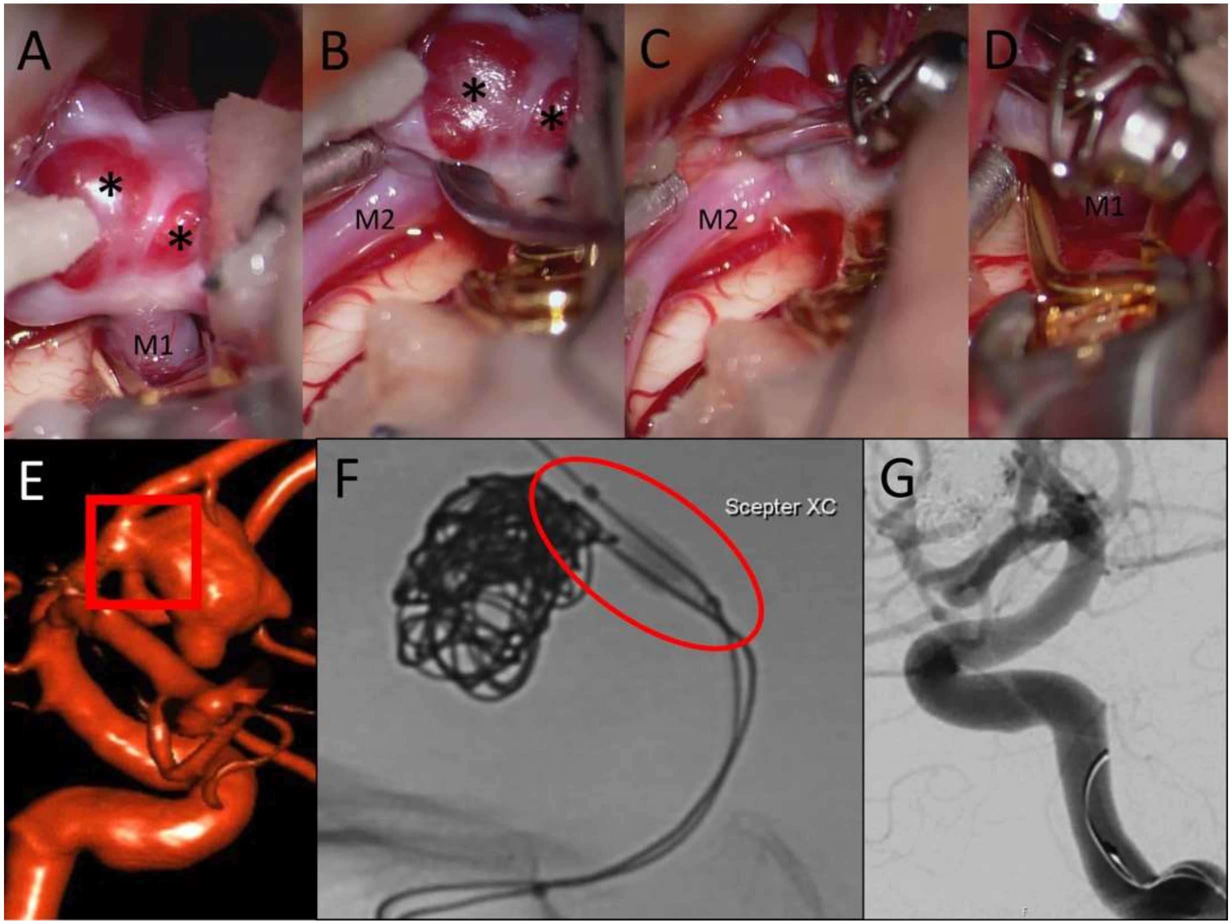

Supplemental Figure 1. Examples of temporary occlusion techniques commonly used in cerebrovascular surgery.

A temporary occlusion clip (gold colour) is applied to the parent artery (M1) of a middle cerebral artery (MCA) aneurysm during surgery (A). This allows safe dissection of the neck of the aneurysm that has a partially fragile wall (marked with *) (B). The temporary occlusion also decompresses the aneurysm, facilitating ligation with a permanent clip (silver colour) (C). Once the permanent clips are in place, the temporary clip occluding M1 is removed and flow in M2 branches verified. The ACom aneurysm with the other A2 segment arising from the aneurysm neck (E) is an example of an endovascular case in which temporary occlusion of the neck region with an endovascular balloon (marked with a red ellipsoid in F) is used to ensure that the coil loops used to embolize the aneurysm are properly placed. Once the aneurysm is packed with coils, the temporary occlusion balloon is deflated and flow in the arterial tree is restored (G).
