# Supplementary material for: Pretreatment with a dual antiplatelet and anticoagulant (APAC) reduces ischemia–reperfusion injury in a mouse model of temporary middle cerebral artery occlusion—implications for neurovascular procedures
Source: Acta Neurochir (Wien). 2024 Mar 15;166(1):137. doi: 10.1007/s00701-024-06017-x (PMC10940479; doi:10.1007/s00701-024-06017-x)
Supplement: Supplementary file 2 — Supplementary file2 (PDF 535 KB) [file 701_2024_6017_MOESM2_ESM.pdf]

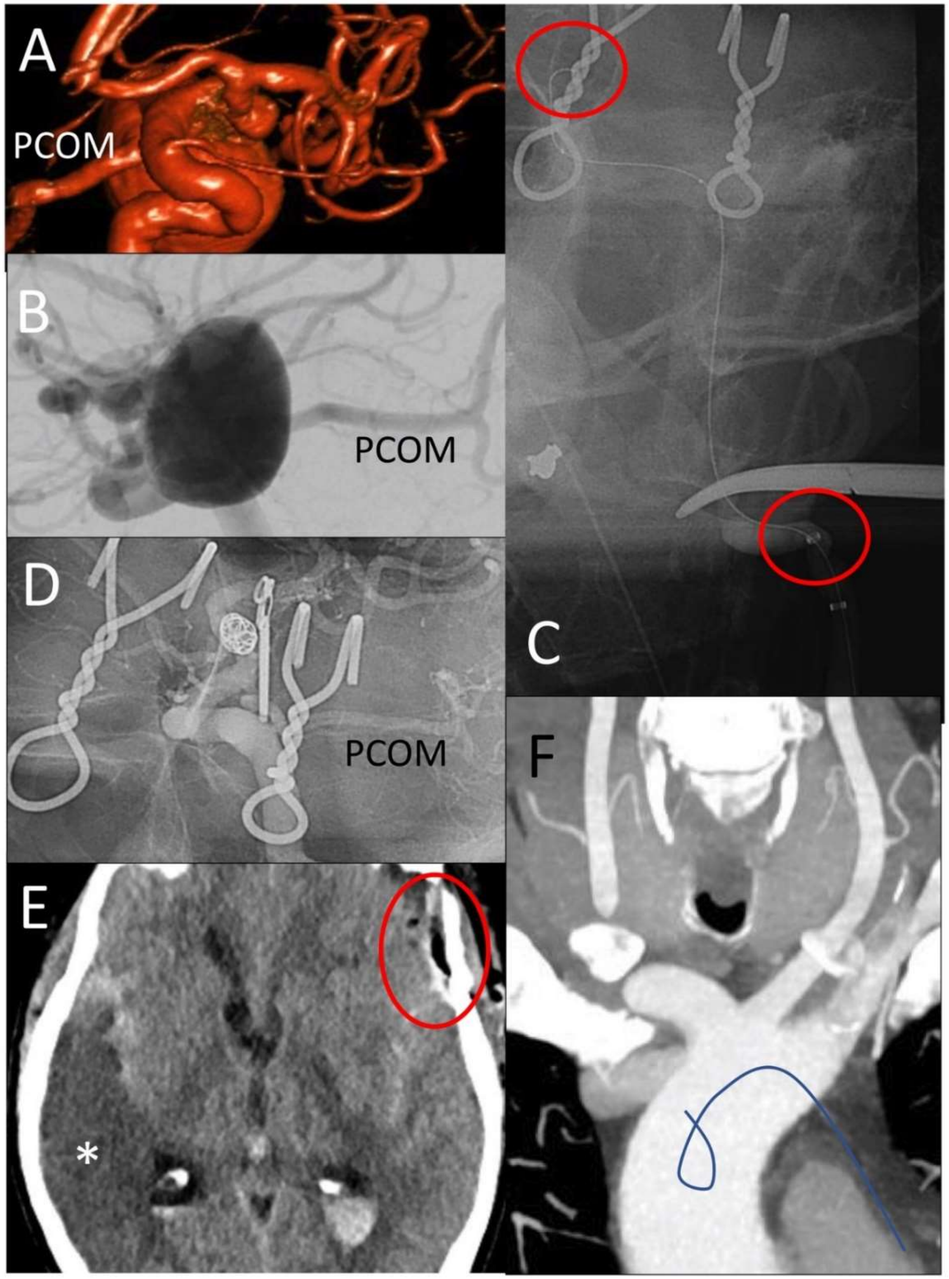

Supplemental Figure 2. Intraprocedural embolization related to catheterization during angiogram.

A large (approx. 2 cm) ruptured posterior communicating artery (PCom) aneurysm (3D digital subtraction angiography from anterior view in A, 2D angiogram from the lateral view in B) with a fetal PCom was ligated as a hybrid operating room procedure with concomitant endovascular balloon occlusion of the internal carotid artery and suction deflation of the large aneurysm (C) to allow successful ligation (D). The patient also had a previously coiled unruptured aneurysm of the anterior choroidal artery visible in D. The procedure was started with placement of the endovascular catheters to the abdominal aorta through standard inguinal puncture, following which the catheters were moved upwards to the left internal carotid artery once the craniotomy and aneurysm exposure had been performed. Probably as a combination of the hypercoagulable state of the patient (observed during the procedure and explained by the aneurysmal subarachnoid hemorrhage) and the time taken to perform the craniotomy and aneurysm exposure, the endovascular catheters started to develop thrombosis despite the use of heparinized saline infusion. This resulted in a contralateral posterior cerebral artery infarction (marked with *, craniotomy marked with red ellipsoid) observed in postoperative CT scan controls (E). An important contributing factor was the patient’s anatomy with a bovine-type aortic arch, i.e., left common carotid artery sharing a common origin with brachiocephalic artery (F), combined with both sides having a fetal PCom supplying the posterior cerebral artery circulation and thus allowing posterior cerebral artery embolization from the carotid circulation.
