# Supplementary material for: Pretreatment with a dual antiplatelet and anticoagulant (APAC) reduces ischemia–reperfusion injury in a mouse model of temporary middle cerebral artery occlusion—implications for neurovascular procedures
Source: Acta Neurochir (Wien). 2024 Mar 15;166(1):137. doi: 10.1007/s00701-024-06017-x (PMC10940479; doi:10.1007/s00701-024-06017-x)
Supplement: Supplementary file 3 — Supplementary file3 (PDF 508 KB) [file 701_2024_6017_MOESM3_ESM.pdf]

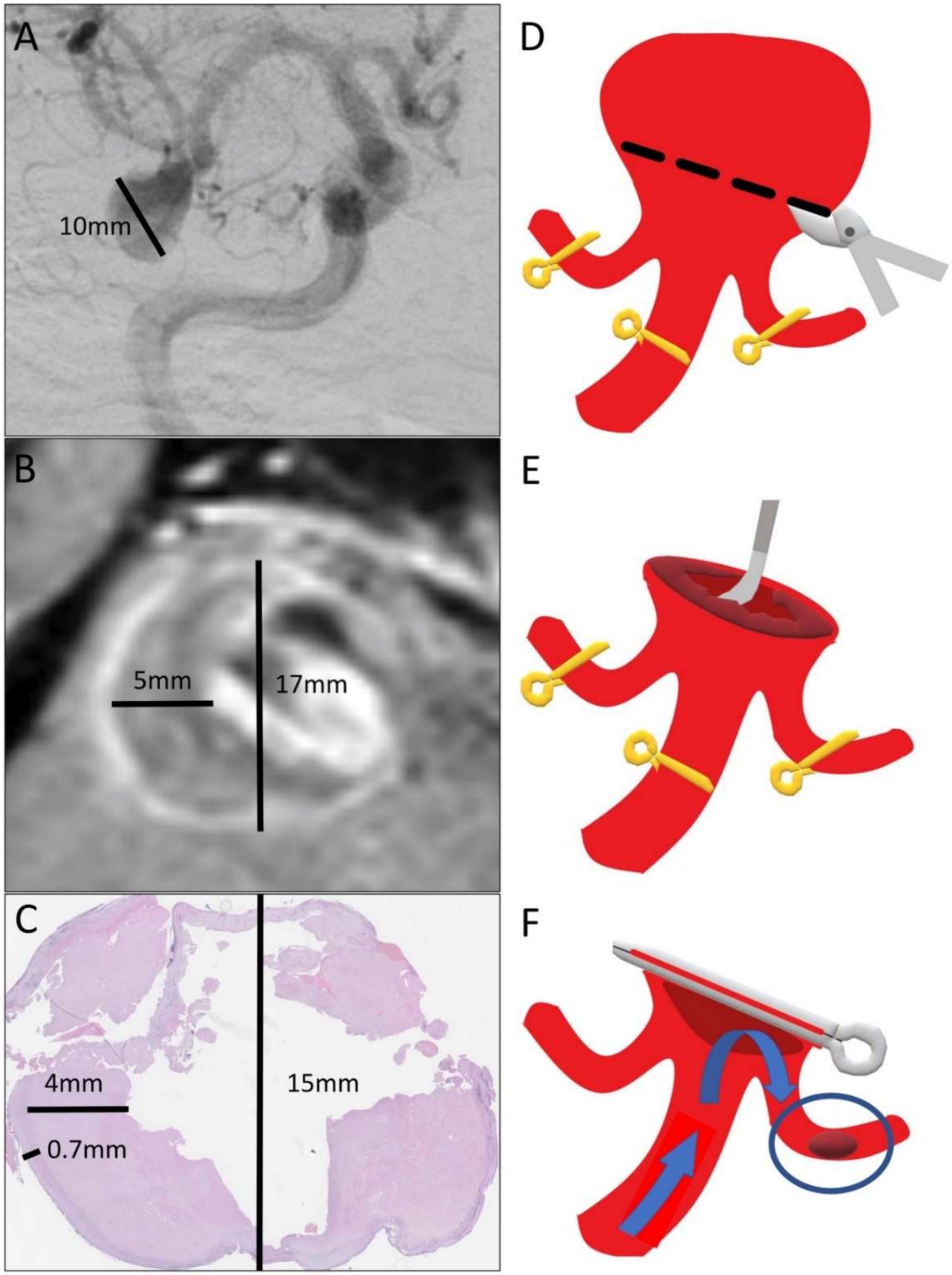

Supplemental Figure 3. Example of a neurosurgical procedure in which APAC could be used to reduce the risk of ischemic complications.

A large middle cerebral artery aneurysm (angiogram in A) that was partially thrombosed (gadolinium enhanced T1 black blood imaging in B) was operated. The intraluminal thrombus layer is visible both in the MRI (5mm) and in the histological cross section (4mm) of the aneurysm dome resected during surgery (C), as well as a size mismatch between the aneurysm filling in angiogram (A, diameter 10mm) and the outer wall of the aneurysm as it appears in MRI (B). To enable ligation of the aneurysm neck, temporary occlusion of the feeding (M1) and branching arteries (M2) was performed, followed by cutting open the aneurysm sac (D) and removal of the luminal thrombus mass (E). Although this intra-aneurysmal thrombectomy procedure softened the neck sufficiently to allow clip ligation, and intraoperative doppler study confirmed the patency of the M2 branches, the thrombogenic surface (in brown) left by the procedure led to delayed embolization of both M2 branches (embolus marked with a blue circle). This resulted in thrombotic occlusion of both M2 branches and the aneurysm neck despite mechanical removal of the emboli with a Fogarty embolectomy catheter. Focal application of a potent anticoagulant to the intraluminal thrombogenic surface of the aneurysm neck could have reduced the risk of emboli or thrombosis.
